# The same heterozygous *Col4A4* mutation triggered different renal pathological changes in Chinese family members

**DOI:** 10.3389/fgene.2023.1180149

**Published:** 2023-05-31

**Authors:** Fengming Zhu, Yueqiang Li, Yuxi Wang, Ying Yao, Rui Zeng

**Affiliations:** ^1^Department of Nephrology, Tongji Hospital, Tongji Medical College, Huazhong University of Science and Technology, Wuhan, China; ^2^Department of Nutrition, Tongji Hospital, Tongji Medical College, Huazhong University of Science and Technology, Wuhan, China; ^3^Key Laboratory of Organ Transplantation, Ministry of Education, NHC Key Laboratory of Organ Transplantation, Key Laboratory of Organ Transplantation, Chinese Academy of Medical Sciences, Wuhan, China

**Keywords:** COL4A4, TNXB, heterozygous gene mutations, hereditary nephritis, renal biopsy

## Abstract

**Background:** Mutations in the collagen components of the glomerular basement membrane (GBM) often lead to hereditary glomerulonephritis. Previous studies have identified that autosomal dominant mutations of *Col4A3, Col4A4* or *Col4A5* are associated with thin basement membrane nephropathy (TBMN), Alport syndrome and other hereditary kidney diseases. However, the genetic mutations underlying other glomerulonephritis types have not been elucidated.

**Methods:** In this study, we investigated a Chinese family with hereditary nephritis using the methods of genetic sequencing and renal biopsy. Genomic DNA was extracted from peripheral blood of the proband and her sister, and subsequently was performed genetic sequencing. They were found to have the similar mutation sites. Other family members were then validated using Sanger sequencing. The proband and her sister underwent renal puncture biopsies, and experienced pathologists performed PAS, Masson, immunofluorescence, and immunoelectron microscopic staining of the kidney tissue sections.

**Results:** Through genetic sequencing analysis, we detected a novel heterozygous frameshift mutation c.1826delC in the *COL4A4* (NM_000092.4) gene coding region, and 1 hybrid missense variation c.86G>A (p. R29Q) was also detected in the *TNXB* (NM_019105.6) gene coding region in several members of this Chinese family. Interestingly, we found that the same mutations caused different clinical features and distinct pathological changes in individual family members, which confirmed that pathological and genetic testing are crucial for the diagnosis and treatment of hereditary kidney diseases.

**Conclusion:** In this study, we found a novel heterozygous mutation in *Col4A4* and co-mutations of the *TNXB* gene in this Chinese family. Our study indicated that the same *Col4A4* mutated variants produced different pathological and clinical changes in different family members. This discovery may provide novel insights into the study of hereditary kidney disease. In addition, new genetic biology techniques and renal biopsy of individual family members are essential.

## Introduction

Hereditary glomerulonephritis is one of the main causes of end-stage renal disease (ESRD). There are currently no effective treatments for this special disease. Therefore, an early and clear diagnosis through genetic testing may have substantial implications for the patient and his or her offspring, and under the proper guidance of a professional doctor, delay the deterioration of renal function.

The majority of hereditary nephritis is inextricably linked to genetic mutations (Mehta and Jim, 2017). Type 4 collagen is the most important component of the glomerular basement membrane and is secreted by podocytes. Mutations in the type 4 collagen gene (*Col4*) and its subunits result in the most common types of hereditary nephritis (Hudson, 2004; Deltas et al., 2013). Previous studies have demonstrated that the mutations in *Col4* and its subunits cause thin basement membrane nephropathy (TBMN) (Cèlia Badenas et al., 2002; Mark Buzza et al., 2003) and Alport syndrome (Bárbara Tazón Vega, 2003), which are the common types of inherited nephritis. Autosomal dominant mutations of *Col-4A3* and/or *A4* lead to the development of TBMN (Hirabayashi et al., 2022). The *Col4A4* gene encodes one of the six subunits of type IV collagen (E D Adamson SEA, 1979). Lemmink, et al. reported the first *Col4A4* mutation in 1996 in TBMN patients (Smeets VVK et al., 1996). After that, more than 20 mutations at different sites of *Col4A4* were described. However, the main mutation sites have not been clarified yet.

The *TNXB* gene encodes a member of the tenascin-X family of extracellular matrix glycoproteins, which is thought to function in collagen organization and extracellular matrix integrity during wound healing (Egging et al., 2007), and its deficiency or mutation has been related to the connective tissue disorder Ehlers‒Danlos syndrome (Schalkwijk et al., 2001; Merke et al., 2013). However, the relationship between *TNXB* mutation and kidney disease has not yet been elucidated.

In this study, we analyzed a Chinese family with hereditary glomerulonephritis and carried out gene mutation analyses using genetic sequencing. According to previous research, we suspected that the same genetic mutation may produce similar pathological changes, however, renal biopsy results challenged this view.

## Materials and methods

### Patients

The research subject is a Chinese family, including the proband, her parents, her son, her sister and the sister’s daughter and son. The proband (II1) is a 46-year-old woman who demonstrated continuous microscopic hematuria and microalbuminuria for more than 10 years and underwent a renal biopsy; chronic glomerulonephritis was confirmed without any deposition of fluorescent material. She then asked for genetic testing. Her mother died of uremia several years ago; therefore, renal biopsy and genetic testing could not be conducted for this individual. Her younger sister showed frequent urination, urgency, and dysuria for 5 years, and her renal biopsy results confirmed IgA nephropathy. Both the proband and her sister showed the mutations in *Col-4A4* and *TNXB* through genetic testing. Her father, her son and her sister’s children did not have any clinical symptoms. We recommended them to take blood and urine samples for primary screening and found that the son of the proband had microscopic hematuria. Then, with the consent of the patient and her family members, we took blood samples from the family members and performed exon sequencing tests. We also advised her son to undergo renal biopsy, which was refused due to a busy work schedule. This study was approved by the Ethics Committee of Tongji Hospital affiliated with Tongji Medical of Huazhong University of Science and Technology. The proband and her family members provided written informed consent to participate in this study.

### Renal biopsy and pathological analysis

The proband and her sister underwent renal biopsy conducted by an experienced nephrologist using a Doppler ultrasound device in the year of 2017. The pathological tissue sections were subjected to Periodic Acid-Schiff (PAS) and Masson staining in our renal pathology laboratory referred to this literature for specific staining methods (Pei et al., 2019). After staining, an experienced pathologist examined the sections using light microscopy, immunofluorescence and electron microscopy.

### Genetic mutation detection

Genomic DNA was extracted from the peripheral blood leukocytes of the proband and family members (Qiagen, Germany) according to the manufacturer’s instructions. The concentration and purity of DNA were measured using a NanoDrop 2000 ultra-microspectrophotometer (Thermo Scientific, United States). High-throughput sequencing was carried out at BGI Genomics (Wuhan, China) with an ABI 3730XL sequencer. Briefly, 1 µg of genomic DNA was used as a template, and specific primers were extended in the presence of DNA polymerase to obtain PCR products. The extension reaction was not terminated until the insertion of a dideoxynucleotide (ddNTP). The amplified products were subjected to 1% agarose gel electrophoresis. Then, the products were purified with a BigDye® XTerminator™ Purification Kit (ABI, United States) and subjected to sequencing with an ABI 3730XL sequencer using a BigDye® XTerminator™ Sequencing Kit (ABI, United States). A heterozygous frameshift mutation was detected in the coding region of the *Col4A4* gene, and a missense mutation was also detected in the coding region of the *TNXB* gene of the proband. Then, the peripheral blood samples from the proband’s sister and other family members were subjected to Sanger sequencing to verify the genetic mutation condition. The pathogenicity of filtered polynucleotides was analyzed using a variety of bioinformatic analysis software (SIFT, (Sorting Intolerant From Tolerant) http://sift.jcvi.org; Mutation Taster, http://www.mutationtaster.org/).

## Results

### Clinical features of patients

**FIGURE 1:**
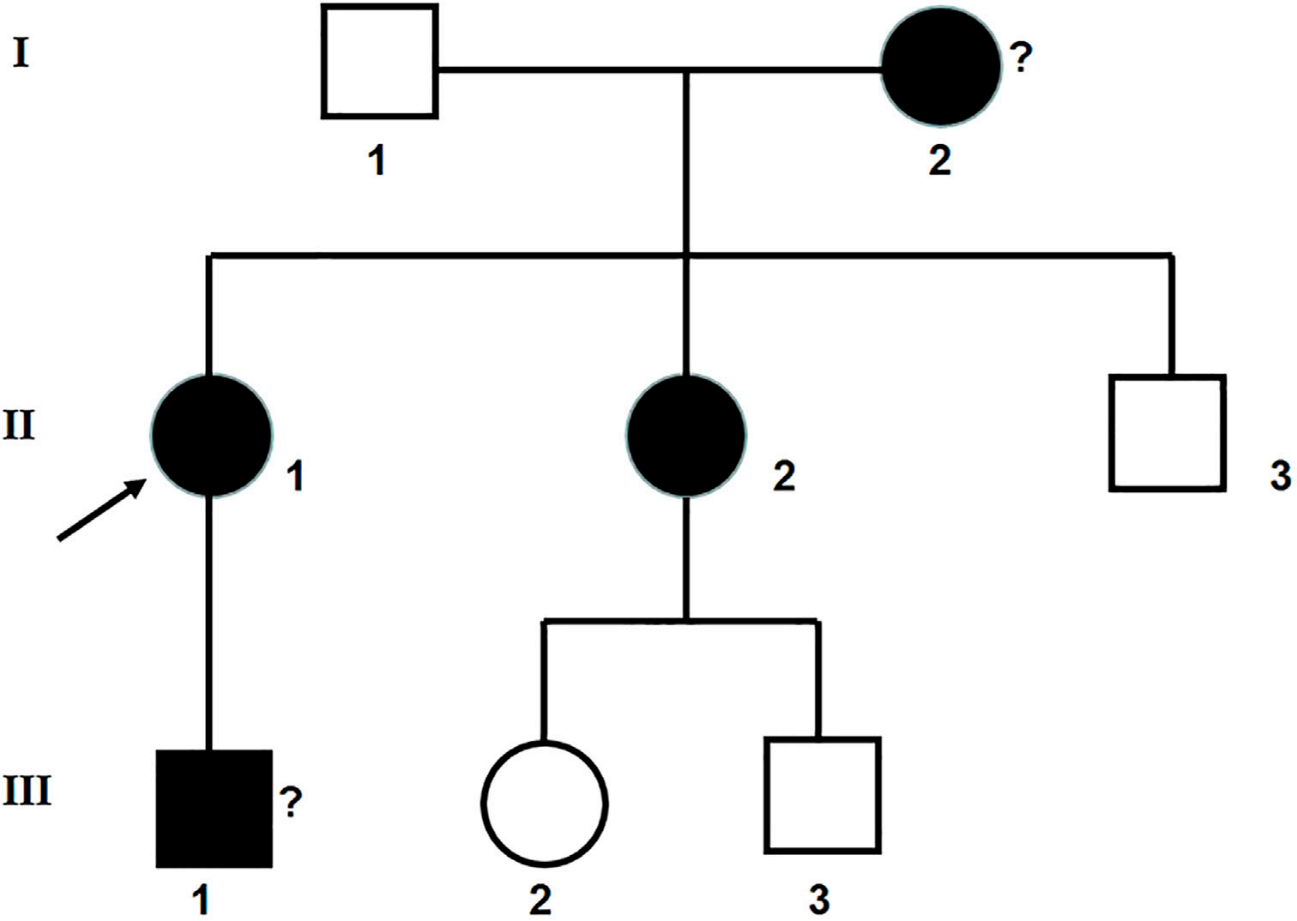
Family diagram for the group with glomerulonephritis. II 1: the proband.

The clinical information was acquired from 7 people in a Chinese family in Hubei Province. The renal biopsy confirmed that the proband and her younger sister had glomerulus nephritis. Her son, who refused to have a renal biopsy, was potentially also a patient according to his routine urine test results. The proband’s mother died of uremia. A family diagram for the group with glomerulonephritis is shown in Figure 1.

The main clinical manifestations of the proband were continuous microscopic hematuria and microalbuminuria for more than 10 years. In the past 3 months, the proband presented with eyelid edema and nocturia (>3 times per night). Microscopic examination of the urine showed that erythrocytes in the urine were mixed, suggesting that the hematuria originated from the glomeruli. The glomerular filtration rate was decreased to 45.9 mL/min/1.73 m^2^, and the level of serum creatinine was 122 μmol/L. The proband’s son had no symptoms. Only laboratory tests revealed microscopic hematuria, and he refused to undergo a renal biopsy.

However, the proband’s younger sister showed different clinical features and was diagnosed with frequent urination, urgency, and dysuria for 5 years. Her routine urine tests showed hematuria and proteinuria, and she was positive for the left renal vein nutcracker syndrome. The routine urine test was negative for hematuria but still showed trace proteinuria after anti-inflammatory treatment. She underwent renal biopsy and was diagnosed with glomerulonephritis.

The test results for rheumatism, immune system and other related factors of the proband and her sister were negative, excluding secondary kidney damage.

The specific clinical characteristics and urine test results of the proband and their relatives are shown in Table 1.

**TABLE 1:** Clinical features, urine/blood tests, kidney biopsy results of proband and carriers in the year of 2017.

Member	Age	Gender	ACR (ug/mg)	Upr (mg/24 h)	URBC	eGFR (mL/min/1.73 m^2^)	Kidney biopsy pathology		
							Light microscopy	Immunofluorescence	Immunoelectron microscopy
Ⅰ 2	Died	Female	—	—	—	—	—	—	—
II 1	46	Female	307.4	407.1	3+	45.9	Similar to FSGS	Negative	Endothelial cells and podocytes swelling, foot process fusion, GBM curled
II 2	40	Female	24.8	255.2	2+	78.5	Mild lesions	IgA 3+	Mild capillary lumen dilation
Ⅲ 1	24	Male	Negative	Negative	2+	101	—	—	—

^a^ URBC, urinary red blood cells; ACR, albumin-to-creatinine ratio; Upr, 24 h urinary albumin excretion; eGFR, estimated glomerular filtration rate; GBM, glomerular basement membrane.

### Pathological features of the proband

Light Microscopy: 3 large fibrocystic crescents with microthrombi, 1 small fibrous-cellular crescent formation, 1 ischemic sclerosis and 3 glomerular sclerosis formations of 11 glomeruli were seen in the renal tissue. Mild and segmental proliferation of mesenchymal cells and stroma were observed in the remaining glomeruli. Several endothelial cells in the glomeruli developed segmental proliferation. Some of the tubules were detached from the tubular base membrane, and some showed multifocal atrophy. Mononuclear cells and lymphocytes infiltrated the renal interstitium multifocally, accompanied by fibrosis. Immunofluorescence: Negative. Immunoelectron microscopy: Capillary loops were irregularly arranged with partial cavity collapse. Endothelial cells showed swelling without obvious proliferation. Podocytes were swollen, with partial vacuolar degeneration and foot process fusion. Some renal tubular epithelial cells were necrotic and exfoliated, and the residual basement membrane was curled. The renal interstitium was scattered with infiltration of inflammatory cells and deposition of collagen fibers. Pathological diagnosis: Focal glomerular lesion with partial crescent formation.

### Pathological features of the proband’s younger sister

**FIGURE 2:**
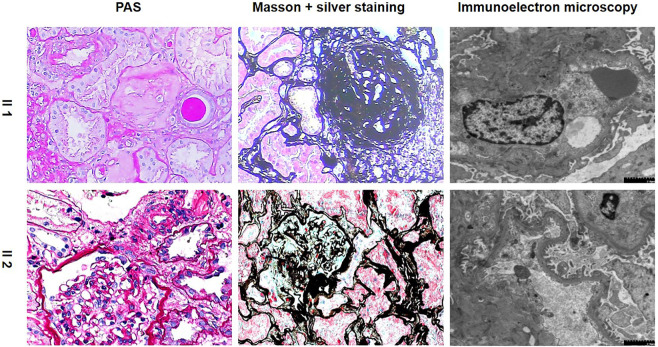
Pathological features of the proband and her younger sister. (PAS, periodic acid-schiff.)

In contrast, the proband’s younger sister showed completely different pathological features. Light Microscopy: 5 of 17 glomeruli showed spherical sclerosis. The capillaries were obviously shrunken. The walls of the capsules were significantly thickened with stratification. The remaining glomeruli were not uniform in size, with a small increase in volume and slight hyperplasia in mesangial cells. Tubulointerstitial lesions were mild-to-moderate, with tubular epithelial cell edema, granular cell degeneration, focal tubular atrophy, basement membrane thickening, and epithelial cell flattening. A small amount of fibrosis and mononuclear cell infiltration and several foam cells were observed in the renal interstitium. Immunofluorescence showed that IgA (+++) was deposited diffusely in the glomerular mesangial area. Immunoelectron microscopy: part of the capillary lumen was dilated. The basement membrane was normal in morphology, and no electron-dense deposits were observed. Most of the foot processes were fused. Pathological diagnosis: IgA nephropathy with mild mesangial proliferative lesions with glomerular sclerosis (5/17), as shown in Table 1; Figure 2.

### Sequencing of *COL4A4* and *TNXB*

**FIGURE 3:**
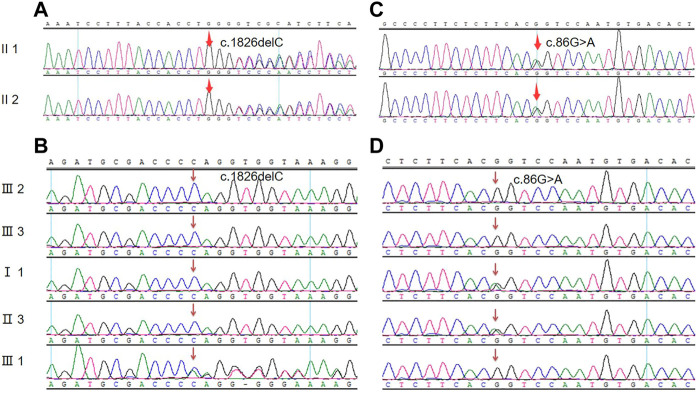
Mutated nucleic acid sequence for the genes of *Col4A4* and *TNXB*. **(A)** Genetic sequencing of the proband and her sister at the splice site of c.1826delC of the *Col4A4* gene. **(B)** Sanger sequencing peak map of other family members at the splice site of c.1826delC of the *Col4A4* gene. **(C)** Genetic sequencing of the proband and her sister at the splice site of c.86G>A of the *TNXB* gene. **(D)** Sanger sequencing peak map of other family members at the splice site of c.86G>A of the *TNXB* gene.

According to the high-throughput sequencing results of the proband in the early stage, a heterozygous frameshift mutation of c.1826delC was detected in the *COL4A4* (NM_000092.4) gene coding region (the corresponding amino acid was p.Pro609Glnfs*44), and 1 hybrid missense variation of c.86G>A (p. R29Q) was also detected in the *TNXB* (NM_019105.6) gene coding region (the corresponding amino acid was p.R29Q). Sanger sequencing of the above sites was performed on the proband and her sister. They possessed the same genetic mutations at the same sites, as shown in Figure 3; Table 2. Her son had only the *Col4A4* mutation. The proband’s father and brother had only a nonsense mutation at the splice site of c.86G>A of the *TNXB* gene. Genetic mutations were not detected in other living family members, as shown in Figure 3; Table 2. The screened nucleotides were subjected to gene mutation analysis (Mutation Taster; SIFT) indicted that the c.1826delC mutation of *Col4A4* was predicted to be pathogenic and the c.86G>A locus of *TNXB* was predicted to be polymorphism (probable harmless) (https://www.mutationtaster.org/MT69/MutationTaster69.cgi). Further enquiries for genetic sequencing data can be directed to the link (https://ngdc.cncb.ac.cn/gsa-human/submit/hra/subHRA006673/finishedOverview).

**TABLE 2:** Genetic screening results of proband and her sister at the splice sites of *COL4A4* and *TNXB* genes.

Member	Gender	Age	Patients or not	*COL4A4* (mutation type; nucleotide change)	*TNXB* (mutation type; nucleotide change^a^)	ACMG criteria
Ⅱ 1	Female	46	Patient	+, Het^a^; c.1826delC	+, Het; c.86G>A	Pathogenic
Ⅱ 2	Female	40	Patient	+, Het; c.1826delC	+, Het; c.86G>A	Pathogenic
Ⅲ 2	Female	17	Normal	–, Het	–, Het	-
Ⅲ 3	Male	10	Normal	–, Het	–, Het	-
Ⅰ 1	Male	67	Normal	–, Het	+, Het; c.86G>A	Benign
Ⅱ 3	Male	45	Normal	–, Het	+, Het; c.86G>A	Benign
Ⅲ 1	Male	24	Patient	+, Het; c.1826delC	–, Het	Likely Pathogenic

^a^ Hom/Het/Hem: Hom, Homozygous mutation; Het, Heterozygous mutation; Hem, Semizygous mutation. Genomic coordinates: the reference sequence is hg19.

*Nucleotide variations are named according to the HGVS (human genome variation society) nomenclature.

ACMG: American college of medical genetics and genomics.

### Follow-up

Prior to receiving the genetic test results, the proband and her sister were given glucocorticoids and immunosuppressants (cyclophosphamide, CTX, cumulative dose of 3 g) treatment for 3 times. The levels of creatinine and urea nitrogen did not obviously change; thus, the intensive treatment was stopped until the genetic test results returned. We then treated the proband with an angiotensin converting enzyme inhibitor (ACEI) to reduce proteinuria, an alpha keto acid to supplement kidney essential amino acids, an oxidized starch with a covered aldehyde group to eliminate small molecule toxins, and Chinese patent medicine in the form of Huangkui capsules and Huaiqihuang granules to reduce the level of albuminuria and hematuria. To date, the eGFR level of the proband has remained stable for 4 years, without substantial anemia, while blood pressure and blood glucose and lipid levels have remained within normal ranges. The proteinuria and hematuria level of the proband’s younger sister were effectively relieved, and the level of creatinine returned to normal. Then, she was treated with an ACEI, Chinese patent medicine in the form of Huangkui capsules, Huaiqihuang granules and Kunxian capsules and other treatments for 5 years. Similar to the proband, her younger sister maintained normal levels of kidney function and urine protein by taking only Candesartan tablets, a type of angiotensin II AT1 receptor antagonist, for 4 years.

**FIGURE 4:**
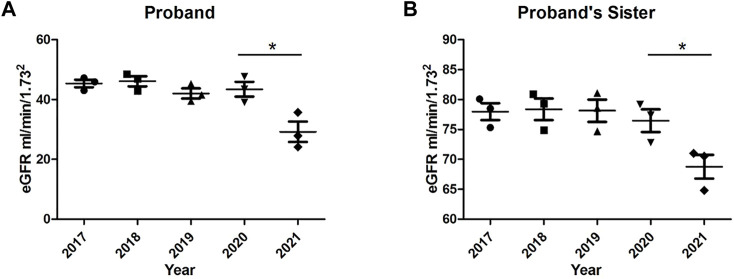
Trends in glomerular filtration rates (eGFR) of the proband and her younger sister over 5 years. **(A)** eGFR of the proband; **(B)** eGFR of the proband’s younger sister. *p < 0.05, *n* = 3–5.

The proband and her sister are reviewed every 2-3 months at our outpatient clinic. Treatment regimen has been adjusted according to renal function and urinalysis results. After 5 years, the renal function of both the proband and her younger sister deteriorated, as shown in Figure 4. Moreover, the proband began to develop mild anemia and elevated blood pressure. We adjusted the treatment plan in hopes of slowing the deterioration rate of renal function.

## Discussion

In this study, we analyzed a Chinese family with hereditary kidney disease through renal biopsy and genetic sequencing technology. We identified a novel variant, *Col4A4* c.1826delC, and a hybrid missense variation in *TNXB* c.86G>A. Interestingly, we found that the same genetic mutations manifested different renal pathological changes through examination of pathological specimens from renal biopsies, in which the proband showed focal glomerular lesions with partial crescent formation, while her sister showed IgA nephropathy with glomerular sclerosis.

Type 4 collagen, which is secreted by endothelial cells and epithelial cells into the extracellular matrix and then self-assembled into a polygonal network structure, is the main component of the GBM extracellular matrix and is encoded by 6 genes (*Col4A1-A6*) [(E D Adamson SEA, 1979); *B V Howard et al. (1976);*(*Khoshnoodi et al., 2008*; *Deng et al., 2016*). Among them, the mutation of the *COL4A4* gene produces an abnormal and incorrectly bound α4 (IV) chain, which enters the triple helix structure of type IV collagen, leading to the instability of the upper molecular structure (Khoshnoodi et al., 2008). Numerous studies have confirmed that mutations in different subtypes of *Col4A3, A4*, and *A5* can lead to *Col4*-associated glomerulopathy (Bárbara Tazón Vega, 2003), including Alport syndrome (AS) (Korstanje et al., 2014; Guo et al., 2017; Du et al., 2022), thin basement membrane nephropathy (TBMN) (Xu et al., 2016; Hirabayashi et al., 2022), and focal segmental glomerulosclerosis (FSGS) (Xie et al., 2014; Wu et al., 2016; Fan et al., 2020).

However, unlike previous studies, we found genetic mutations of *Col4A4* in some members of this family, but neither the proband nor her relatives had any pathological and clinical manifestations of the related diseases such as AS and TBMN. According to the electron microscopy results, the GBM of the proband and her sister only presented minor lesions. In genetic sequencing, we detected an autosomal recessive mutation in the Col4A44 gene. This mode of inheritance usually requires two pathogenic mutations to cause the clinical phenotype. Therefore, we speculate that the single pathogenic locus mutation detected in this study only results in a small fragment base abnormality or an in-frame 3-fold shift, which may form partially retained functional truncated proteins, which are not yet sufficient to cause abnormalities in the morphology and structure of the triple helix of type IV collagen. According to the light microscopy results, the proband’s lesions tended to be focal glomerular lesions with crescents but without obvious sclerosis. Her sister showed IgA nephropathy with mild mesangial proliferative lesions with glomerular sclerosis. The presence of microscopic hematuria in the son of the proband does not exclude the possibility of IgA nephropathy. None of the family members presented sensorineural hearing abnormalities, deafness or early-onset renal failure. We speculate that this paradox may be due to the fact that heterozygous mutations trigger a relatively mild phenotype than homozygous mutations because normal *α4* (*IV*) chains are still produced.

We found that the proband and her sister had different renal pathological changes. There was no deposition of fluorescent substances in the kidney tissue of the proband, while IgA deposition was detected in the mesangial area of her younger sister. Thus, we assumed that the different fluorescence deposition states represented different kidney pathological changes. Stapleton and others found candidate variants in 10 families and identified a likely pathogenic variant in *COL4A5* in one family and a variant of unknown significance (VUS) in *COL4A3* in another (Stapleton et al., 2020), which confirmed the heterogeneity of IgA nephropathy (Stapleton et al., 2020) and provided evidence that while a proportion of patients with renal IgA deposits may carry pathogenic variants of known kidney disease-related genes such as the *Col4* gene, others may not (Stapleton et al., 2020). In our study, we found that *Col4* mutation can lead to focal segmental glomerulosclerosis, which is consistent with previous literature reports (Xie et al., 2014; Wu et al., 2016).

Previous studies have detected *Col4A3/A4/A5* mutations in patients with familial IgA nephropathy (Savige et al., 2021). In a recent study, Li Zhu et al. detected 31.1% of genotypic mutations in the Col4A3/A4/A5 genes in patients with IgA nephropathy with thinned GBM lesions (IgAN-tGBM) and in 0.19% patients with sporadic IgA nephropathy (Yuan et al., 2023). In this study, the pathological diagnosis of the proband’s sister (II-2) confirmed IgA nephropathy, and genetic testing suggested a mutation in the *Col4A4* and *TNXB* gene. However, no significant lesions were seen in the glomerular basement membrane. Other family members do not match the diagnostic criteria for IgA nephropathy, which was considered that II-2 might be a patient with sporadic IgA nephropathy accompanied by *Col4* gene mutation without tGBM.

Previous research showed that mutations in the *COL4A4* gene can cause benign familial hematuria (OMIM# 141200) (Cèlia Badenas et al., 2002), which is autosomal dominant; in addition, mutations in the *TNXB* gene (encoding tenascin-X, an extracellular matrix protein) can cause vesicoureteral reflux type 8 (OMIM#: 615,963) (Gbadegesin et al., 2013), which is autosomal dominant as well. In our study, the proband and her sister demonstrated benign familial hematuria, the same variation in *Col4A4* was identified in both individuals, which was consistent with known reports (Bárbara Tazón Vega, 2003; Yang et al., 2019). Furthermore, we detected a hybrid missense variation in *TNXB* c.86G>A in four members of this family. However, none of them showed abnormalities during kidney color Doppler ultrasound, such as bladder ureteral reflux. Dr. Deborah P. Merke and others demonstrated that biallelic *TNXB* variants caused Ehlers‒Danlos syndrome in patients with congenital adrenal hyperplasia (Merke et al., 2013; Watanabe et al., 2021; Marino et al., 2022). All of the patients had skin hyperextensibility and significant joint hypermobility. Joint laxity was extreme and some patients had a history of joint dislocations, chronic arthralgias, and chronic tendinitis and/or bursitis. There were no clinical manifestations of skin hyperextensibility or significant joint hypermobility in the proband and her family members in this study. The severity of the disease depended on the carrying capacity of the *TNXB* variant, as evidenced by the allelic heterogeneity of the cohort (Chen et al., 2016). Combined with this finding, we speculated that the *TNXB* mutation identified in our study may be a monoallelic hybrid missense mutation, and whether it is common or unique in frequency is unknown at this time. Further research is needed to investigate this mutation and its manifestations.

A large number of studies have shown that patients with hereditary kidney diseases do not undergo the active intensive treatment because of their genetic mutations (Mehta and Jim, 2017; Boyer et al., 2021). In this study, as the renal biopsy report showed partial crescent formation, the proband underwent intensive treatment with glucocorticoids + CTX in the early stages of disease, aiming to suppress immune and inflammatory responses, and thereby reducing the formation of crescents. According to the results of the 5-year follow-up observation, the proband’s renal function remained stable without obvious anemia, which was attributed to the early intensive treatment. The immunofluorescence kidney biopsy results of the proband’s sister, who also received early glucocorticoid + CTX treatment showed a large number of IgA deposits in the glomeruli. Renal function was stable, which was due to the intensive and powerful immunosuppressive therapy during the early stage. After 3 cycles of treatment, hematuria and albuminuria were obviously relieved. At this point, genetic testing confirmed hereditary glomerulonephritis, and glucocorticoid and immunosuppressive therapy was discontinued.

The limitations of this study are as follows: 1. the son of the proband did not undergo renal biopsy; 2. Immunofluorescence staining was not finished at the renal specimens for different *Col4* subtypes. In the future, we will continue to suggest the son of the proband to undergo a renal biopsy and use the frozen sections to conduct immunofluorescence staining for *Col4* subtypes as a way to confirm the conclusion that the collagen mutation in this study is pathogenic. There is currently no effective treatment for hereditary kidney disease. In the future, kidney function may continue to deteriorate in the proband, and her family members may pass on their *Col4A4* mutation to subsequent generations. We will continue to search for new treatments for them. The proband’s son is now of child-bearing age, and we suggested him to consider selecting healthy embryos through new reproductive technologies to avoid the transmission of mutated genes from generation to generation (Bergmann, 2017).

In conclusion, we identified a Chinese family with similar genetic mutations but different pathological changes and clinical phenotype by genetic testing and renal puncture biopsy pathology. This study enriches the genetic spectrum of hereditary kidney diseases and also provides new ideas for the diagnosis and treatment of these diseases.

## Data Availability

The data presented in the study are deposited in the Genome Sequence Archive (GSA)-OMIX repository, accession number OMIX004157.
